# Oxygen Consumption and Oxygen Pulse Patterns in Two Pediatric Patients With Pulmonary Hypertension and Different Clinical Responses: A Case Report

**DOI:** 10.1155/crpe/6697634

**Published:** 2026-05-23

**Authors:** Allegra J. VanderWilde, Kimberley G. Miles, Kristian C. Becker, Paul J. Critser, Michelle Cash, Melissa Magness, Wayne A. Mays, Russel Hirsch, Adam W. Powell

**Affiliations:** ^1^ Department of Pediatrics, University of Cincinnati, Cincinnati, Ohio, USA, uc.edu; ^2^ The Heart Institute, Cincinnati Children’s Hospital Medical Center, Cincinnati, Ohio, USA, cincinnatichildrens.org; ^3^ Division of Cardiology, Nemours Children’s Health, Wilmington, Delaware, USA, nemours.org

**Keywords:** cardiopulmonary exercise testing, congenital heart disease, oxygen pulse, oxygen pulse plateau, pulmonary hypertension

## Abstract

Two patients with repaired congenital heart disease (CHD) and CHD‐associated pulmonary arterial hypertension (PAH) are presented to illustrate the utility of cardiopulmonary exercise testing in this population. Novel markers used include the oxygen pulse curve (O_2_pulse) as an initial marker of PAH–CHD disease progression. Both patients had an abrupt decrease in their peak oxygen consumption on serial exercise testing. In addition to the decrease in the peak oxygen consumption, Patient 1 also had a fall in the O_2_pulse curve during progressive exercise and an episode of concerning symptoms, prompting an alteration medication management. Patient 2 had a similar drop in peak oxygen consumption without a fall in the O_2_pulse curve, and this patient remained stable on their current medical therapies. The overall aim of this case study is to highlight the utility of cardiopulmonary exercise testing in patients with CHD‐associated PAH and showcase the utility of exercise testing in optimizing clinical management.

## 1. Introduction

Pulmonary arterial hypertension (PAH) has previously been considered an absolute contraindication to cardiopulmonary exercise testing (CPET) [1]; however, more recent research demonstrates that this form of testing can be safely administered in pediatric populations [2, 3]. While more centers are performing surveillance CPET in youth with PAH–congenital heart disease (CHD), there remains a gap in our understanding of the expected changes in CPET data for these patients. CPET has important prognostic utility in individuals with acquired and CHD; specifically, the slope of minute ventilation to expired carbon dioxide (VE/VCO_2_ slope; measure of ventilatory efficiency) is a known predictor of morbidity and mortality in CHD patients within the next 5 years [4]. The role of CPET in pediatric PAH remains unclear [5, 6]. Oxygen pulse (O_2_pulse) represents the amount of oxygen consumed per heartbeat (mL/beat) and is the ratio of peak oxygen consumption (VO_2_) divided by heart rate (HR), serving as an indirect measure of stroke volume [7]. In typical individuals without cardiac disease, the O_2_pulse increases to ∼ 50% of the peak VO_2_ and then plateaus, mimicking the expected timing of stroke volume and HR augmentation during exercise (Figure 1(b)) [7]. Although changes in O_2_pulse have not been specifically studied in pediatric PAH patients, changes in stroke volume may serve as an important indicator of overall cardiac function. Here, we present two cases of patients with PAH–CHD (WHO Group 1). While undergoing regular surveillance CPET, these patients showed a significant decrease in peak VO_2_ on serial testing compared to prior testing. Further evaluation revealed different etiologies in each case, and assessment of the O_2_pulse was a useful early indicator of the differing underlying pathophysiologies, as well as directly impacted their clinical care, influencing medical management. These cases are presented to show that O_2_pulse behavior rather than peak values was a potential marker of clinical change for these patients and should be considered in other similar patients.

FIGURE 1(a) demonstrates a graphical display of HR (heart rate) and oxygen pulse (O_2_pulse; VO_2_/HR) (*y*‐axis) versus time (*x*‐axis) from rest of peak exercise. (b) demonstrates different types of O_2_pulse patterns. In a typical patient, the O_2_pulse will increase until roughly halfway through exercise, then plateau. In a highly trained athlete, the O_2_pulse will continue climbing until peak exercise. In Patient 1, the O_2_pulse briefly climbs and then deteriorates until 8 min of exercise, suggesting reduction in stroke volume at near maximal exercise effort. At that time, the O_2_pulse begins to fall until exercise is completed.

**Figure:**
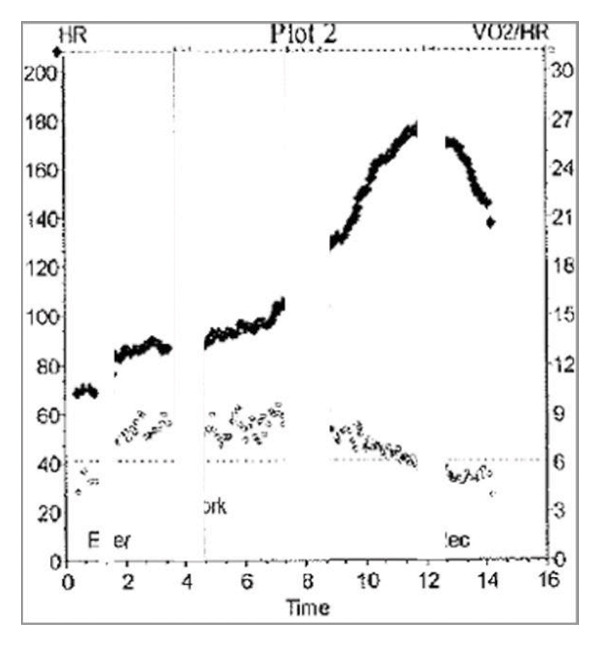
(a)

**Figure:**
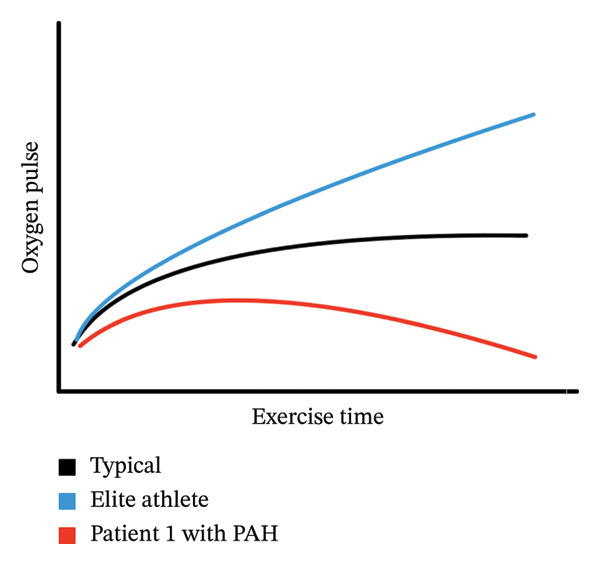
(b)

## 2. Case 1

The first patient was a 15‐year‐old female with a history of D‐transposition of the great arteries (D‐TGA) with a ventricular septal defect (VSD) s/p arterial switch and VSD closure at 3 years of age. Her residual cardiac disease includes PAH–CHD (WHO Group 1, Functional Class I), a small residual restrictive VSD, and a bicuspid neoaortic valve with normal systolic function.

At age 8, she completed her first surveillance CPET at our institution to assess her functional capacity and EKG response. At that time, she was managed with dual therapy (tadalafil and macitentan). She had no cardiac symptoms (Functional Class I) and was active in typical age‐appropriate activities without exercise limitations. Her CPET demonstrated low‐normal peak VO_2_ (744 mL/min measured; 79% of predicted) with a normal peak HR (183 bpm measured, 91.5% of predicted). Her O_2_pulse at peak exercise was 4 mL/beat (86% of predicted).

Her next CPET two years later was grossly unchanged, including a normal peak VO_2_ (83% of predicted), normal peak HR (180 bpm, 90% of predicted), and a normal O_2_pulse at peak exercise of 5.7 mL/beat (91% of predicted). Her VE/VCO_2_ slope at this time was elevated (35, normal < 32).

On repeat CPET at 12 years of age, she had a small interval decrease in her peak VO_2_ (74% of predicted) with a normal peak HR (174 bpm measured, 87% of predicted). Notably, her O_2_pulse, while having a close to normal peak value (85% of predicted), demonstrated an abnormal early plateau followed by a fall in O_2_pulse during the latter stages of exercise (Figure 1(a)). Her VE/VCO_2_ slope at this time was also elevated (peak 39) and had increased from her prior CPET (previously 35). There was no noticeable change in her echocardiogram at this visit compared to the prior one.

The subsequent year, her CPET demonstrated continued limitation with a reduction in her peak VO_2_ (44% predicted, previously 74% predicted) despite a normal peak HR (175 bpm measured, 88% predicted). During this study, her O_2_pulse decreased further (50% of predicted), and the curve demonstrated a similar pattern to the previous test with early flattening followed by a decrease prior to peak exercise. Further, at the end of her test during recovery, she experienced a presyncopal episode on the bike. Her echocardiogram on the same day showed worsening of RV hypertension with preserved function. Due to these changes on her CPET and echocardiogram, her clinical team recommended she undergo reassessment by cardiac catheterization. While her hemodynamics were largely unchanged (pulmonary vascular resistance [PVR]: 8.0 iWU; cardiac index [CI]: 3.73 L/min/m^2^), she was commenced on selexipag (an oral prostanoid agonist). After 6 months of therapy, she reported improvement in exercise tolerance without presyncopal symptoms. Repeat exercise testing is pending.

## 3. Case 2

The second patient was a 17‐year‐old female with a history of perimembranous VSD s/p repair at 8 years of age. Shortly after repair, she was diagnosed with PAH–CHD (WHO Group 1), which was managed with dual therapy (tadalafil and ambrisentan).

Her first CPET at 13 years of age was performed to establish her exercise baseline. At this time, she reported no cardiac symptoms (Functional Class I). Her CPET demonstrated a normal peak VO_2_ (85% of predicted) with a mildly decreased peak HR (155 bpm, 77% of predicted). Oxygen saturation was normal with exercise. Two years later, she underwent a repeat CPET with similar findings, with a normal peak VO_2_ (86% of predicted) and a mildly decreased peak HR (157 bpm, 78% of predicted). Her O_2_pulse at peak exercise was normal on both studies (> 100% of the predicted value).

With assessment 3 years later, her CPET demonstrated an interval decrease in her functional capacity to a peak VO_2_ of 44% of predicted. Her peak O_2_pulse also decreased to 59% of predicted and her peak HR was similar to the previous test (150 bpm; 75% of predicted). While remaining normal, her VE/VCO_2_ slope increased from 24 to 30 on this test.

During the most recent CPET (17 years of age), her O_2_pulse curve showed early flattening without a decrease in peak exercise (Figure 2(a)). During the CPET, she demonstrated no adverse symptoms, nor did she feel that her usual functional capacity had changed between exercise tests. Given the interval change in peak VO_2_ and slope of the O_2_pulse curve between CPETs, her clinical team recommended repeat cardiac catheterization, which she underwent 6 months later. Her results from the catheterization showed stable hemodynamics with similar PVRi (3.9 iWU; 3.4 iWU 4 years prior). During this catheterization, her CI was measured in the normal range, but lower than previously (3.6 L/min/m^2^ compared to 4.1 L/min/m^2^ on prior catheterization at age 12). She also had another CPET repeated 6 months later, and her peak VO_2_ improved slightly to 57% with no symptoms reported. No changes in medication management have been made.

FIGURE 2Graphical display of the heart rate (HR) and oxygen pulse (O_2_pulse; VO_2_/HR) (*y*‐axis) versus time from rest to peak exercise (*x*‐axis). (a) demonstrates the early plateau of the O_2_pulse that stays approximately unchanged to peak exercise. (b) demonstrates graphically the difference between O_2_pulse curves for Patients 1 and 2.

**Figure:**
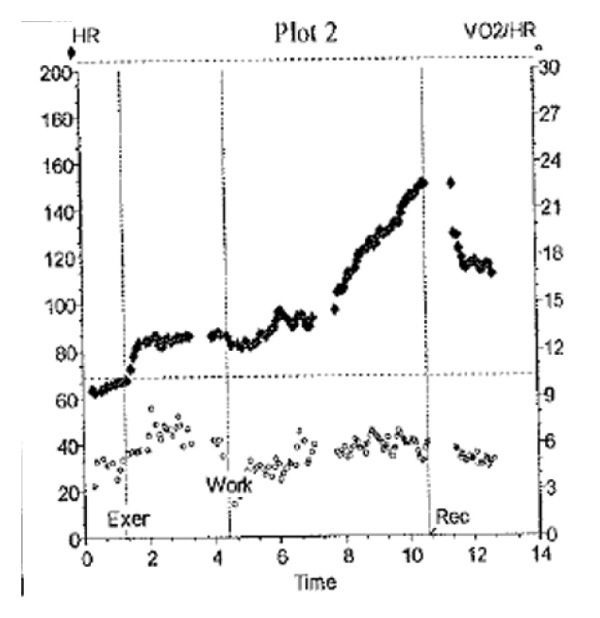
(a)

**Figure:**
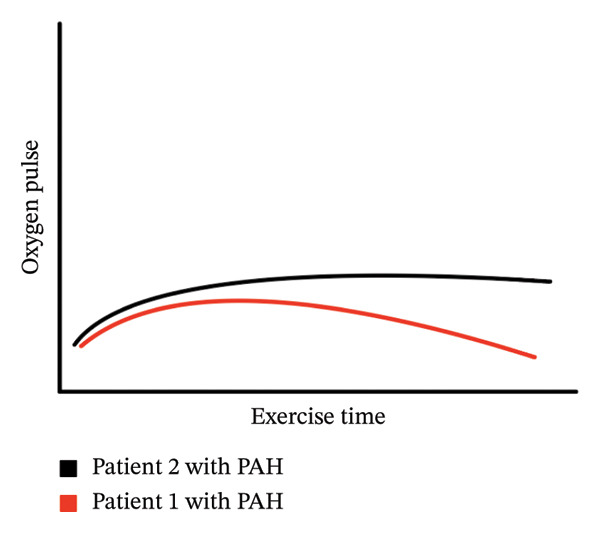
(b)

## 4. Discussion

This report describes two cases of patients with CHD who had developed PAH (WHO Group 1). Utilizing multiple exercise metrics, including O_2_pulse, HR, and peak VO_2_, we determined potential areas of concern in cardiac performance despite both patients remaining clinically asymptomatic. Because both patients underwent regular surveillance CPET, testing frequency was adjusted based on the change in peak predicted VO_2_, and care was modified in Patient 1. The key exercise testing concern for Patient 1 was the decrease in both the O_2_pulse curve and absolute peak O_2_pulse, suggesting reduced ability to augment stroke volume despite maximum metabolic demand during peak exercise, which ultimately manifested as near syncope. Further clinical evaluation demonstrated stable severe PAH physiology, but with worsening symptoms during exercise, escalation of pulmonary vasodilator therapy was felt necessary. In contrast, the second patient demonstrated a significant decrease in peak VO_2_ between interval CPETs, as well as early flattening of the O_2_pulse curve, but without a fall in the O_2_ pulse curve during peak exercise (Figure 2(b)). This suggested adequate stroke volume capacity during increased metabolic demand, which was further supported by this patient’s lack of clinical symptoms during exercise.

The O_2_pulse curve represents the ratio of VO_2_ to HR over the entire period during exercise, and if assuming a stable oxygen extraction during exercise, allows an estimation of stroke volume [7, 8] (Figure 1(b)). In Patient 1, the early flattening and then fall in the O_2_pulse curve during heavy/peak exercise (with a normal peak HR for age) raises concerns for a drop in stroke volume during progressive exercise related to underlying worsening PAH and exercise‐induced alterations in PVR. The typical response to exercise should be a fall in PVR during exercise, but exercise has previously been used to unmask pulmonary hypertension [9]. In the setting of pulmonary vasculopathy with elevated afterload, the normal fall in PVR does not occur, resulting in a marked increase in pulmonary artery pressure. The mismatch in the pressure that the right ventricle (RV) must overcome leads to RV–pulmonary artery uncoupling, especially at the transition from moderate to high‐intensity exercise [10]. This further results in impaired ventricular–ventricular interactions and reduced left ventricular filling, exacerbating the negative impact on stroke volume and cardiac output at higher levels of exercise. This manifests as a fall in O_2_pulse near peak exercise (Figure 2) [10–12].

A fall in the O_2_pulse curve has been shown to predict negative outcomes in multiple pathologies where cardiac stroke volume is deranged. In adults without overt cardiac disease, a fall in O_2_pulse predicts overall cardiovascular morbidity [13], myocardial ischemia [14], and coronary arterial stenosis severity [15]. An early plateau and subsequent decline in the O_2_pulse curve have also been shown to correlate with stroke volume, cardiac output, and right ventricular pressure in adult patients with tetralogy of Fallot [16]. In patients with hypertrophic cardiomyopathy, abnormal O_2_pulse behaviors during exercise have been shown to help identify more advanced disease irrespective of whether left ventricular outflow obstruction is present. In that report, the authors recommended evaluating O_2_pulse kinetics following CPET to risk‐stratify patients with hypertrophic cardiomyopathy [17]. In patients with aortic valve stenosis, an observed fall in O_2_pulse is associated with mortality [18]. Our case report further supports sequential measurement of O_2_pulse curve characteristics in patients with PAH to determine the impact of changes on outcome. O_2_pulse curve characteristics that follow this observed trend, with an early plateau and subsequent decline, may be an important early CPET marker of change in cardiac output and may predict clinical deterioration. Further study of this phenomenon in pediatric PAH patients is thus warranted.

In addition to the different clinical outcomes observed after CPET, these cases highlight concerning trends based on the fall in peak VO_2_, which prompted care changes and treatment escalation in one patient. Similar to other patients with CHD, trending peak VO_2_ could be a useful tool in this PAH population [19–21]. The subtle change in O_2_pulse curve data was an early marker of eventual peak VO_2_ change and was associated with overall clinical decline (decrease in exercise capacity and worsened hemodynamics).

There are inherent limitations when generalizing results of a case study to clinical populations. For one, both patients had different underlying CHD subtypes, PAH severities, and hemodynamics. Additionally, O_2_pulse remains an indirect measure of stroke volume on CPET. Additional investigation is needed to confirm that stroke volume falls during exercise in these patients.

In summary, there is potential value in serial CPET performance in pediatric patients with PAH. Abrupt drops in peak VO_2_ should raise concern for clinical deterioration, similar to other populations. Additionally, an early plateau and then a fall in the O_2_pulse curve during peak exercise may represent a decrease in effective stroke volume and predict a decline in functional exercise reserve, although this should be confirmed in larger studies.

## Funding

No funding was received for this manuscript.

## Ethics Statement

All patient identifiers including name, initials, date of birth, and hospital identification numbers have been omitted from this report to protect the patients’ anonymity. This case report was exempt from Institutional Review Board review. No written consent has been obtained from the patients as there are no patient identifiable data included in this case report.

## Conflicts of Interest

The authors declare no conflicts of interest.

## Data Availability

The data that support the findings of this study are available on request from the corresponding author. The data are not publicly available due to privacy or ethical restrictions.
